# The effect of rehabilitation training based on brain-computer interface on limb function in stroke patients: a systematic review and meta-analyses

**DOI:** 10.3389/fneur.2026.1750875

**Published:** 2026-07-01

**Authors:** Ziqiu Zheng, Changyue Zhang, Mengjiao Lv, Chunting Qian, Ge Gao, Saisai Wang, Rujing Zhu, Jing Yang, Chengwei Zhang, Haiyan Xu

**Affiliations:** 1 Rehabilitation Teaching and Research Section, School of Nursing, Jilin University, Changchun, Jilin, China; 2 Department of Clinical Medicine, North Henan Medical University, Zhengzhou, Henan, China; 3 Department of Gynecology I, Women’s Hospital School of Medicine Zhejiang University Jilin Hospital, Changchun, Jilin, China; 4 Department of Anesthesiology, The Second Hospital of Jilin University, Changchun, Jilin, China

**Keywords:** brain computer interface training, limb function, meta-analyses, rehabilitation, stroke

## Abstract

**Background:**

Characterized by high incidence rate, high disability rate, high mortality rate and high recurrence rate, stroke has become the second leading cause of death globally and the primary cause of adult disability. Though traditional rehabilitation methods have played a significant role in post-stroke functional recovery, their therapeutic efficacy is limited. In recent years, brain-computer interfaces (BCI) have advanced rapidly and are being used more frequently in rehabilitation training for stroke patients.

**Objective:**

This systematic review and meta-analyses aimed to systematically assess the effect of brain-computer interface-based rehabilitation training on limb function in patients after stroke, and further investigate the efficacy differences among various types of brain-computer interfaces and treatment protocols.

**Methods:**

The search strategy was conducted in 5 databases (PubMed, Scopus, Web of Science, Embase, and Cochrane Library databases) from inception to August 29, 2025. The studies that explored the impact of BCI combined with rehabilitation on limb function in stroke patients was mainly focused. A meta-analyses was performed using a random effects model, with the weighted mean difference (WMD) and 95% confidence intervals (CIs) as the effect sizes.

**Results:**

Twenty-seven RCTs, including 23 that reported changes in upper limb function and four that reported changes in lower limb function, were included. The results showed that the training based on BCI significantly improved FMA-UE (Fugl-Meyer Assessment upper - extremity) [WMD = 3.50, 95% CI: (2.09, 4.90), *p* < 0.001] and FMA-LE (Fugl-Meyer Assessment lower -extremity) [WMD = 2.59, 95% CI: (1.94, 3.23), *p* < 0.001], compared with the control group.

**Conclusion:**

The combined therapy was effective in improving the limb function of patients. BCI-based training might be a reliable rehabilitation program to improve limb function.

**Systematic review registration:**

https://www.crd.york.ac.uk/PROSPERO/view/CRD420251038208.

## 1 Introduction

Stroke, a common acute neurological disorder caused by cerebrovascular lesions (1), has become the leading cause of adult disability worldwide (2). It accounts for approximately 15 million new cases each year, and about 5 million of those patients are left with permanent disability (3). Motor dysfunction is particularly concerning as it is the most common and serious sequela following a stroke. About 60–80% of patients are left with upper limb dysfunction (4), and 30–50% are left with lower limb dyskinesia (5). This not only impedes patients’ ability to grasp and walk, but also seriously affects their quality of life and ability to participate in society (6–8), resulting in a heavy psychological and social burden (9). Therefore, restoring limb motor function has become the focus of stroke rehabilitation.

The core pathological mechanism underlying motor dysfunction following a stroke is the disruption of neural pathways (10), which prevents motor signals from upper motor neurons from reaching lower motor neurons. This disrupts the integration and regulation of motor sensory input, resulting in impaired innervation of the affected limbs and ultimately manifesting as reduced motor control and impaired motor function. Traditional rehabilitation refers to a set of conventional methods that promote motor function recovery after a stroke, including physical therapy, occupational therapy, motor relearning training, constraint-induced movement therapy (CIMT), mirror therapy, and so on. Its mechanisms of action include: (1) promoting synaptic remodeling and cortical functional reorganization to reestablish coordinated control between the cerebral cortex and peripheral muscles; (2) activating intact neural pathways to restore partial motor function through compensatory mechanisms (11, 12); (3) improving local microcirculation and muscle tone, and regulating neurotransmitter release to suppress abnormal signals (13, 14); and (4) utilizing visuomotor coupling or high-intensity task-specific training to strengthen specific neural pathways. Through repetitive active or passive training, these methods can promote the recovery of motor function to some extent (15). However, their feedback mechanisms rely heavily on the therapist’s visual assessment and manual adjustments, lacking real-time closed-loop detection and regulation of neural signals. This prevents interventions from precisely guiding central plasticity toward functional recovery, resulting in poor rehabilitation outcomes. Moreover, traditional rehabilitation has significant limitations regarding training intensity, patient engagement, intervention timing, rehabilitation duration, and treatment outcomes (16).

As a technology for regulating the central nervous system and enhancing neural plasticity, brain-computer interfaces (BCI) can overcome the limitations of traditional rehabilitation and offer new possibilities for restoring limb function in stroke patients (17). BCIs bypass damaged peripheral nerve-muscle pathways, enabling direct interaction between the brain and external devices (18, 19). Its fundamental characteristic resides in the employment of high-temporal-resolution real-time signal acquisition and multidimensional feature decoding methodologies to translate neural electrical activity into actionable commands. This facilitates individuals in interacting with the external environment without dependence on speech or limb movements, thereby establishing a “central-peripheral-central” closed-loop rehabilitation paradigm. BCIs promote motor function recovery by temporally coupling motor intentions with sensory inputs, inducing changes in neural plasticity within motor networks, and thereby facilitating the functional reorganization of motor control networks (20–22). BCIs can be classified into three categories based on the location of signal acquisition: invasive, semi-invasive, and non-invasive. Non-invasive brain-computer interfaces are the most widely adopted due to their safety, portability, and cost-effectiveness. There are numerous methods for recording brain signals in non-invasive BCI systems, among which EEG is the most commonly used. Common sources of EEG signals include motor imagery (MI), steady-state visual evoked potentials (SSVEP), and P300 (23, 24). BCIs are typically integrated with end-effectors such as functional electrical stimulation, exoskeletons, and visual feedback (25–27), which are responsible for the actual execution of movements or sensory output.

Although BCIs combined with conventional rehabilitation have shown promise in restoring limb function after stroke (28–30), existing studies on their efficacy have yielded conflicting results. Some studies have shown that BCIs can significantly improve upper limb motor function, as well as enhance lower limb balance and gait symmetry, through real-time neurofeedback and multimodal stimulation. However, some trials failed to demonstrate a statistically significant difference between the BCI and control groups. For instance, one study reported no statistically significant improvement in the FMA-UE score in the BCI group relative to conventional therapy. Similarly, a recent meta-analyses indicated that BCI training did not result in significantly greater improvements in upper limb motor function compared to conventional robot training (31). Furthermore, previous meta-analyses primarily focused on upper limb function while neglecting improvements in lower extremity function (32). Therefore, this meta-analyses aims to systematically review existing randomized controlled trials to comprehensively evaluate the effects of BCI-based training on limb function in post-stroke patients. Meanwhile, further subgroup analyses was conducted to explore the factors that might affect the therapeutic effect, with the aim of providing a more comprehensive evidence-based basis for clinical practice.

## 2 Methods

We conducted this systemic review and meta-analyses according to the latest Preferred Reporting Items for Systematic Reviews Meta-analyses (PRISMA) guideline (33). The study protocol has been registered in Prospective Register of Systematic Reviews (PROSPERO) with the registration number: CRD420251038208.

### 2.1 Search strategy

As of August 29, 2025, qualified studies were retrieved in five databases (PubMed, Web of Science, EMBASE, Cochrane Library and Scopus). The search strategy is created by using various combinations of predefined search terms related to stroke, Brain-computer interface, limb function, and randomized controlled trials, along with Boolean operators (AND, OR, NOT). For more details on search terms and search strategies, see Supplementary Table 1. In addition, we manually searched the reference lists of the included studies and relevant reviews to ensure that no relevant studies were overlooked.

### 2.2 Inclusion criteria

We included studies that met the following criteria. (1) All the participants included in the studies were adults meeting the clinical diagnostic criteria for stroke or diagnosed with stroke by MRI (Magnetic Resonance Imaging) or CT (Computed Tomography) (34), and suffering from limb motor dysfunction; (2) The experimental group received BCI-based training, including BCI-FES, BCI-robot, BCI-visual feedback training; (3) Control groups received sham BCI training or conventional rehabilitation training; (4) The study must report upper limb or lower limb functional levels; (5) The study type was randomized controlled trial, reporting the mean and standard deviation before and after intervention or differences; (6) The literature was published in English.

### 2.3 Exclusion criteria

The exclusion criteria included: (1) Studies involving subjects with lock-in syndrome, or traumatic brain injuries; (2) Second-hand unoriginal research (reviews, meta-analyses, letters, reports, conference abstracts); (3) Cannot extract relevant data.

### 2.4 Quality assessment

The quality assessment of the included studies was conducted using the Risk of Bias Tool for Randomized Trials (ROB 2.0) tool, with two authors conducting the assessment independently. In case of disagreement, the two authors discussed to reach a consensus or consulted a third party. The assessment of ROB for eligible studies considered five key items: (1) bias in the randomization process (2) bias in deviating from established interventions (3) bias in missing outcome data (4) bias in outcome measurement (5) bias in selective reporting of outcomes. The evaluation results were classified as “low risk,” “some concern,” or “high risk.” When all risks were “low risk” or there was only one “some concern,” the overall evaluation was classified as “low risk.” If there was more than one “some concern” but no “high risk,” the overall evaluation was classified as “some concern.” If any domain was “high risk,” the overall evaluation was classified as “high risk.”

### 2.5 Data extraction

Data extraction was conducted by two independent authors. When there was a dispute, consult a third party. The extracted information from each study included the first author’s name, year of publication, country, participant age, sample size, stroke phases, interventions and control details, measured outcomes, external feedback, and follow-up evaluation after interventions. If the mean and SD of change scores were shown in the articles, they were extracted. If not explicitly stated, change scores were calculated using the following formula, based on the principles of the Cochrane Handbook for Systematic Reviews of Interventions: Mean_change = Mean_post – Mean_pre, SD_change = √(SD^2^_pre + SD^2^_post – 2 × r × SD_pre × SD_post). In cases where studies reported median and interquartile range, we converted these values to mean and SD estimates using the transformations: Mean ≈ Median, standard deviation = (Q3 – Q1)/1.35.

### 2.6 Statistical analyses

Review Manager (Version 5.4. Cochrane Collaboration, Oxford, England) and Stata software (version 17.0 SE Stata Corp LP, College Station, TX, USA) were used to analyze data. The included outcome measures were all continuous variables. Outcome measures with the same measurement methods and units were represented by weighted mean difference (WMD) and its 95% confidence interval (CI). For outcome measures with different methods or units, standardized mean difference (SMD) and its 95% CI were used. Since all the literature outcome measures included in this study were evaluated using the same scale, WMD and 95% CI were defined as the effect size of this meta-analyses. The Cochrane Q-test and *I*^2^ value were used to evaluate the heterogeneity of the combined results in the study. A *p*-value < 0.10 or an *I*^2^ value > 50% was considered to indicate significant heterogeneity, and a random-effects model will be used. To explore potential sources of clinical heterogeneity in the included studies, subgroup analyses were conducted based on age, disease duration, severity of sports injury, BCI external feedback method, and training intensity. Sensitivity analyses was used to determine the impact of a single study on the overall results. Publication bias was assessed by funnel plots and the Egger’s test. When the funnel plot exhibited asymmetry and the *p*-value of Egger’s test <0.05, it was indicative of potential publication bias. And the trim-and-fill method would be used to recalculate the combined effect size, and *p* < 0.05 would be considered statistically significant.

## 3 Results

### 3.1 Literature search

**Figure 1:**
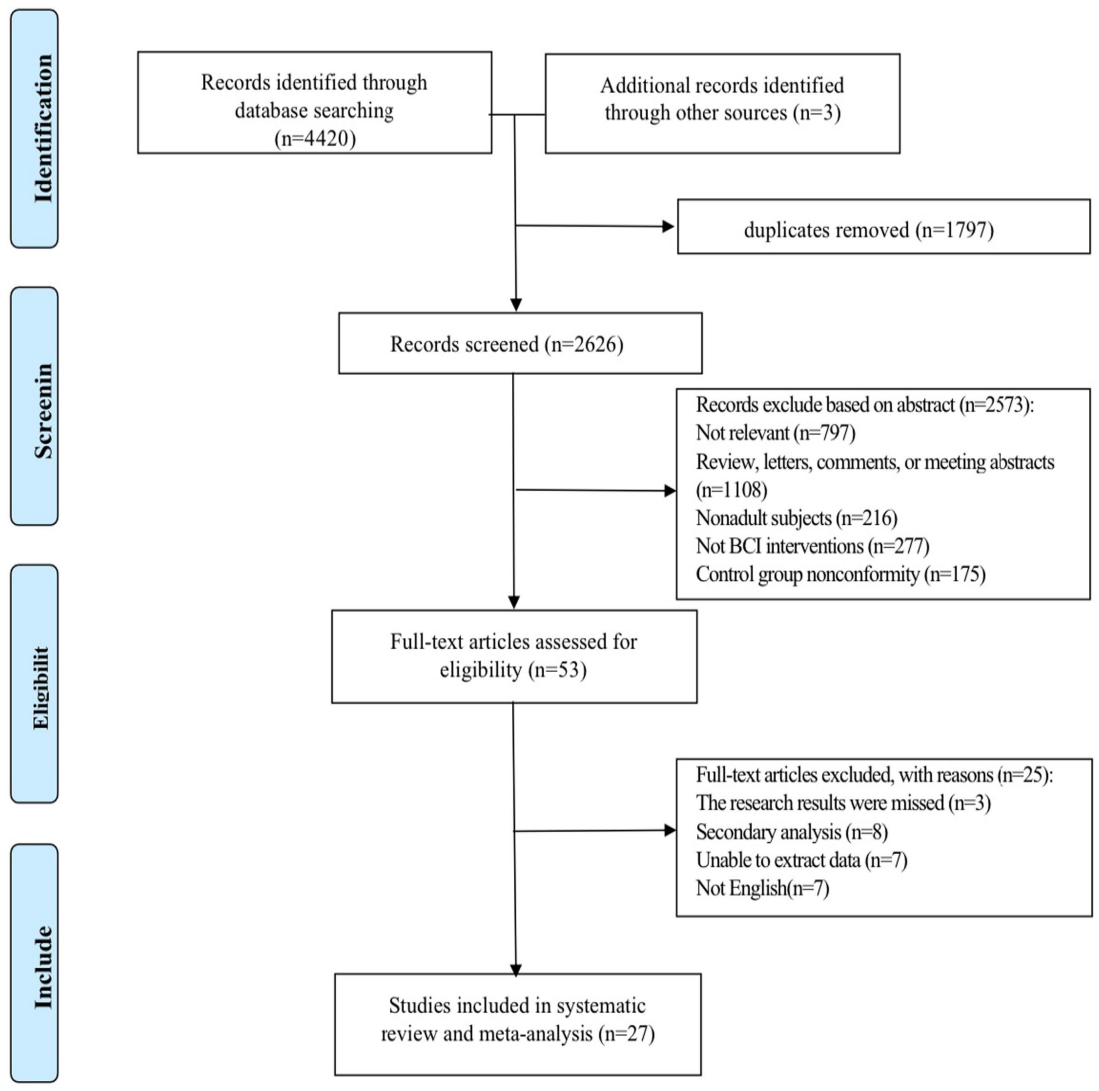
Flow diagram for the identification and exclusion of studies. BCI, Brain-computer interfaces.

The search strategy initially retrieved 4,420 potentially relevant results and found 3 additional studies through other sources, mainly from the reference lists of the retrieved materials articles. After removing duplicates in the text, 2,626 studies were included, and 2,573 were excluded after the title and abstract screening. The following reasons were given for exclusion: (1) The outcome indicators of the study did not report limb function; (2) The studies were reviews, letters, commentaries, meeting abstracts; (3) The study subjects were neither adults nor stroke patients; (4) The experimental group did not adopt BCI; and (5) The control group did not meet the requirements. After reading the entire text, 26 references were excluded for the following reasons: (1) The research results were missed; (2) The studies were secondary analyses; (3) Articles where data cannot be extracted, and (4) Not published in English. Finally, 27 articles were included in the meta-analyses. The flowchart of the literature search and detailed reasons for exclusion were shown in the Figure 1.

### 3.2 Characteristics of included studies

27 studies published between 2013 and 2025 were included (25–30, 35–55), involving 1,023 participants. These studies were conducted in 3 different regions. 20 studies were carried out in Asia (26–29, 35–40, 42–48, 52–54), five in Europe (25, 30, 49, 51, 55) and two in North America (41, 50). Participant ages ranged from 27.5 to 69.5 years. The intervention lasted from 2 to 8 weeks, among which 9 trials lasted less than 4 weeks (37, 39, 43, 45, 46, 48, 52, 53, 55), 12 trials lasted for 4 weeks (26–28, 30, 35, 38, 41, 42, 47, 49, 51, 54), and 5 trials lasted from 5 to 8 weeks (25, 29, 36, 44, 50). 10 articles include stroke patients in the subacute phase (26–28, 43–45, 48, 49, 53, 54), and 11 studies focused on the chronic phase (25, 29, 30, 35, 36, 39, 41, 42, 47, 51, 55). According to the feedback methods of BCI, it could be classified into three types, among which there were 9 articles focusing on BCI-FES (25, 26, 41, 42, 44, 45, 47, 52, 55), 15 articles focusing on BCI-Robot (27–29, 30, 35–39, 43, 46, 50, 51, 53, 54), and 3 articles focusing on BCI-Visual feedback (40, 48, 49). Six studies were followed up, with the follow-up periods ranging from 2 weeks to 6 months (see Table 1).

**Table 1:** Main information extracted from included studies.

Study	Country	N	Age	Sex (M/F)	Time from stroke onset	Stroke phases	Experimental interventions	Control interventions	training intensity	Outcome measures	Follow-up
Ang et al. (2014)(36)	Singapore	E: 6	E: 54.0 ± 8.9	E: 4/2	E: 285.7 ± 64.0 d	Chronic	BCI-HK + con-rehab	HK: HK + con-rehab	60 min /d, 3 d/w,	FMA-UE	6 w
		C: 8/7	C: 51.1 ± 6.3/58.0 ± 19.3	C: 6/2;4/3	C: 454.4 ± 109.6 d			SAT: con-rehab	6 w, 18 sessions		18 w
Ang et al. (2015) (35)	Singapore	E: 11	E: 48.5 ± 13.5	E: 9/2	E: 383.0 ± 290.8 d	Chronic	BCI-Manus robot	Manus robot	90 min/d, 3 d/w,	FMA-UE	8 w
		C: 14	C: 53.6 ± 9.5	C: 7/8	C: 234.7 ± 183.8 d				4 w,12 sessions		
Biasiucci et al. (2018) (25)	Switzerland	E: 14	E: 56.4 ± 9.9	E: 6/8	E: 39.8 ± 45.9 m	Chronic	BCI-FES + con-rehab	Sham FES + con-rehab	60 min/d, 2 d/w,	FMA-UE	6–12 m
		C: 13	C: 59.0 ± 12.4	C: 10/3	C: 33.5 ± 30.5 m				5 w, 10 sessions	MRC	
										MAS	
										ESS	
Chen et al. (2020) (28)	China	E: 7	E:41.6 ± 12.0	E: 7/0	E: 3.1 ± 1.7 m	Subacute	BCI-exoskeleton + con-rehab	Sham BCI + con-rehab	19.5 min/d, 3 d/w,	FMA-UE	—
		C: 7	C: 52.0 ± 11.1	C: 4/3	C: 3.9 ± 1.5 m				4 w, 12 sessions		
Cheng et al. (2020) (29)	China	E: 5	E: 62.4 ± 4.7	E: 3/2	E: 476.8 ± 302.0 d	Chronic	BCI-SRG + con-rehab	SRG + con-rehab	90 min /d, 3 d/w,	FMA-UE	6 w
		C: 5	C: 61.4 ± 4.5	C: 2/3	C: 890.2 ± 257.2 d				6 w, 18 sessions	ARAT	18 w
Curado et al. (2015) (30)	Germany	E: 16	E: 46.6 ± 13.4	E: 5/2	E: 5.6 ± 3.9 y	Chronic	BCI-orthosis + con-rehab	Sham BCI + con-rehab	60 min/d, 5 d/w,	FMA-UE	—
		C: 14	C: 49.9 ± 13.0	C: 6/1	C: 5.5 ± 6.1 y				4 w, 20 sessions		
Frolov et al. (2017) (37)	Russia	E: 55	E: 56.9 ± 12.9	E: —	E: 8.4 ± 6.9 m	Subacute and chronic	BCI-exoskeleton + con-rehab	Sham BCI	30 min/d, 5 d/w,	FMA-UE, ARAT	—
		C: 19	C: 59.1 ± 12.0	C: —	C: 10.9 ± 7.9 m				2 w, 10 sessions		
Fu et al. (2023) (38)	China	E: 30	E: 55.93 ± 11.05	E: 23/7	E: 77.5 (33.8, 175.3) d	Unclear	BCI-hand robot + con-rehab	Con-rehab	30 min/d, 5 d/w,	FMA-UE	—
		C: 31	C: 59.00 ± 14.49	C: 25/6	C: 64.0 (37.0, 150.0) d				4 w, 20 sessions		
Guo et al. (2022) (39)	China	E: 10	E: 60.2 ± 9.3	E: 9/1	E: 12.5 ± 7.1 m	Chronic	BCI-SRG + con-rehab	Robotic: SRG	60 min/d, 5 d/w,	FMA-UE	12 w
		C: 10/10	C: 53.5 ± 8.3/56.9 ± 6.1	C: 8/2;8/2	C: 10.9 ± 7.9 m			Control: con-rehab	2 w, 10 sessions	WMFT	
										MAS	
Hu et al. 2021 (40)	China	E: 7	E: 44.9 ± 7.5	E: 4/3	E: 8.27 ± 1.98 m	Unclear	BCI-visual feedback + con-rehab	Con-rehab	30 min/d	FMA-UE	
		C: 5	C: 60.4 ± 16.8	C: 4/1	C: 7.80 ± 1.78 m					ARAT	
										BI	
										MSS	
Kim et al. (2016) (41)	USA	E: 15	E: 59.1 ± 8.1	E: 6/9	E: 8.3 ± 2.0 m	Chronic	BCI-FES + con-rehab	Con-rehab	30 min/d, 3 d/w,	FMA-UE	—
		C: 15	C: 59.9 ± 9.8	C: 6/9	C: 7.8 ± 1.8 m				4 w, 12 sessions	MAL	
										MBI	
										ROM	
Lee et al. 2022 (42)	Korea	E: 13	E:55.2 ± 11.6	E:4/9	E: 7.5 ± 1.6 m	Chronic	BCI-FES + con-rehab	FES + con-rehab	30 min/d, 5 d/w,	FMA-UE	—
		C: 13	C: 58.3 ± 9.2	C: 6/7	C: 8.3 ± 2.0 m				4 w, 20 sessions	WMFT	
										MAL	
										MBI	
Li et al. (2014) (44)	China	E: 7	E: 66.3 ± 4.9	E: 5/2	E: 2.2 ± 1.8 m	Subacute	BCI-FES + con-rehab	FES + con-rehab	60–90 min/d, 3 d/w,	FMA-UE	—
		C: 7	C: 67.1 ± 6.0	C: 6/1	C: 2.8 ± 2.0 m				8 w, 24 sessions	ARAT	
Li et al. (2022) (43)	China	E: 12	E: 43.8 ± 14.7	E: 12/0	E: 4.0 (2.0, 11.3) m	Subacute	BCI-exoskeleton + con-rehab	Con-rehab	60 min/d, 5d/w,	FMA-UE	2 w
		C: 12	C: 55.0 ± 12.2	C: 12/0	C: 4.3 ± 2.60 m				2 w, 10 sessions	WMFT, MBI	
Liu et al. (2023) (45)	China	E: 30	E: 52.5 ± 10.59	E: 22/8	E: 52.5 (45.0, 59.3) d	Subacute	BCI-FES + con-rehab	FES + con-rehab	20 min/d, 5d/w,	FMA-UE	—
		C: 30	C: 53.0 ± 15.56	C: 19/11	C: 18.0 (9.8, 23.0) d				3w, 15 sessions	WMFT	
										MBI	
Ma et al. (2023) (46)	China	E: 20	E: 50.90 ± 12.64	E: 16/4	E: 5.90 ± 2.99 m	Unclear	BCI-exoskeleton + con-rehab	Con-rehab	40 min/d, 5d/w,	FMA-UE	—
		C: 20	C: 58.30 ± 11.23	C: 15/5	C: 6.45 ± 3.38 m				2 w, 10 sessions		
Miao et al. (2020) (47)	China	E: 8	E: 48.8 ± 16.7	E: 6/2	E: 18.3 ± 10.9 m	Chronic	BCI-FES + con-rehab	Con-rehab	16 min/d, 3 d/w,	FMA-UE	—
		C: 8	C: 50.3 ± 17.1	C: 6/2	C: 11.1 ± 5.0 m				4 w, 12 sessions		
Mrachacz-Kersting et al. (2016) (55)	Denmark	E: 13	E: 46.31 ± 12.51	E: 11/2	E: 15.38 ± 6.2 m	Chronic	BCI-FES + con-rehab	Sham BCI + con-rehab	3 days	FMA-LE	—
		C: 9	C: 55.22 ± 10.63	C: 8/1	C: 18 ± 4.47 m					10WMT	
Mihara et al. (2013) (48)	Japan	E: 10	E: 56.1 ± 7.9	E: 8/2	E: 146.6 ± 36.2 d	Subacute	BCI-visual feedback + con-rehab	Sham BCI + con-rehab	20 min/d, 3 d/w,	FMA-UE	2 w
		C: 10	C: 60.1 ± 8.5	C: 4/6	C: 123.4 ± 38.3 d				2 w,6 sessions	ARAT	
Pichiorri et al. (2015) (49)	Italy	E: 14	E: 64.1 ± 8.4	E: —	E: 2.7 ± 1.7 m	Subacute	BCI-virtual feedback + con-rehab	MI + con-rehab	60 min/d, 3 d/w,	FMA-UE	—
		C: 14	C: 59.6 ± 12.7	C: —	C: 2.5 ± 1.2 m				4 w, 12 sessions	MRC	
										MAS	
Rodríguez-García et al. (2025) (50)	Mexico	E: 10	E: 47.8 ± 15.796	E: 7/2	E: —	Subacute and chronic	BCI-robot + con-rehab	Sham BCI + con-rehab	60 min/d,5d/w	FMA-UE	—
		C: 9	C: 55.778 ± 14.99	C: 10/9	C:—				1.5 m		
Ramos-Murguialday et al. (2013) (51)	Germany	E: 16	E: 49.3 ± 12.5	E: 9/7	E: 66.0 ± 45.0 m	Chronic	BCI-orthosis + con-rehab	Sham BCI + con-rehab	60 min/d, 5 d/w,	FMA-UE	6 m
		C: 16	C: 50.3 ± 12.2	C: 9/5	C: 71.0 ± 72.0 m				4 w, 20 sessions	MAL	
										AS	
										GAS	
Wang et al. (2024) (26)	China	E: 150	E: 60 (52–67)	E: 110/40	E: 15 (8–21) d	Subacute	BCI-FES + con-rehab	Con-rehab	30 min/d, 5 d/w,	FMA-UE	2 m
		C: 146	C: 58 (52–66)	C: 115/31	C: 13 (8–1) d				4 w, 20 sessions	ARAT	
										WMFT	
										MAS	
										IADL	
Wu et al. 2019 (27)	China	E: 14	E: 62.9 ± 10.6	E: 9/5	E: 2.1 ± 0.3 m	Subacute	BCI-exoskeleton + con-rehab	Con-rehab	60 min/d, 5 d/w,	FMA-UE	—
		C: 11	C: 64.8 ± 7.2	C: 9/5	C: 2.0 (1.5, 3.0) m				4 w, 20 sessions	ARAT	
										WMFT	
Luo et al. (2024) (52)	China	E: 32	E: —	E: 23/9	E:—	Acute	BCI-FES + con-rehab	Con-rehab	60 min/d	FMA-LE	—
		C: 32	C: —	C: 19/13	C:—				2w, 20 sessions	FAC	
										MBI	
Yuan et al. (2021) (53)	China	E: 16	E: 62.0 (56.8–65.2)	E: 11/5	E: 6.5 (2.8–10.2)w	Subacute	BCI-Robot + con-rehab	Con-rehab	6d/w, 2w	FMA-LE	—
		C: 14	C: 65.5 (62.5–69.8)	C: 6/8	C: 7.5 (4.2–11.5)						
Zhao et al. (2022) (54)	China	E: 14	E: 50.1 ± 11.1	E: 13/1	E: 47.1 ± 53.2 d	Subacute	BCI-robot	shambci	6d/w, 4w	FMA-LE	
		C: 14	C: 56.1 ± 11.5	C: 12/2	C: 27.5 ± 18 d						

E, Experimental group; C, Control group; M, Male; F, Female; BCI, Brain-Computer interface; FES, Functional electric stimulation; con-rehab, Conventional rehabilitation; m, Months; w, week; d, Days; SAT, Standard Arm Therapy; FMA-UE, Fugl-Meyer assessment-upper extremity; ARAT, Action research arm test; WMFT, Wolf motor function Test; MBI, modified Barthel index; IADL, Instrumental Activity of Daily Living; MAS, modified Ashworth scale; AS, Ashworth scale; MRC, Medical Research Council Scale; MAL, Motor Activity Log; GAS, Goal Attainment Scale; ESS, European Stroke Scale; FMA-LE, Fugl-Meyer Assessment-Lower Extremity; 10WMT, 10-meter Walking Test.

### 3.3 Risk of bias assessment

**Figure 2:**
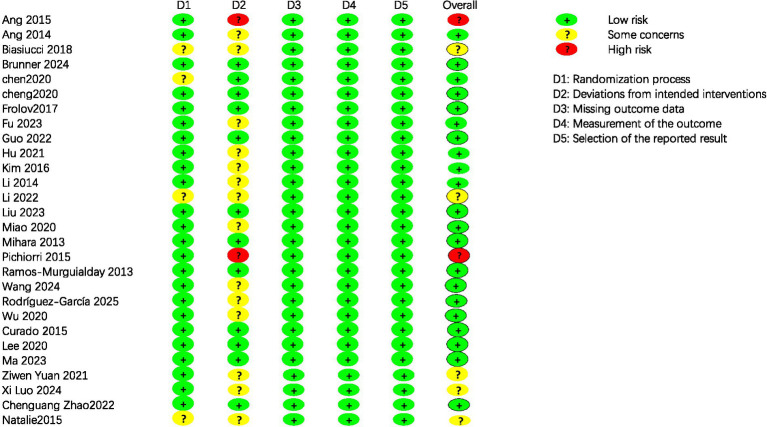
Quality assessment of included studies using the Cochrane collaboration’s tool.

The results of the risk of bias assessment were shown in Figure 2. The study of Ang and Pichiorri’s were evaluated as “high-risk” because the allocation method was unknown. Three studies only mentioned randomness during randomization but did not describe the hidden allocation, and thus were identified as “low-risk.” In addition, all other studies reported random allocation methods, among which 20 studies were rated as “some concerns” because they did not specify whether the allocation sequence was hidden. Inadequate blinding is a major source of risk for study bias. Most studies depend on blinding the assessors, given that it is challenging to blind both participants and the therapists administering the intervention. There were six studies reported a double-blind approach, 15 studies reported a single-blind approach. In five studies, although a single-blind method was used, it was ensured that the intervention provider would not affect the outcome. Four studies reported subject attrition or dropout, while the rest did not. The outcome measurements and the selection of reporting results for all studies were unbiased. Overall, 24 studies were classified as “low risk,” one as “some concerns,” and two as “high risk.”

### 3.4 Meta-analyses and subgroup analyses results

#### 3.4.1 Effect of rehabilitation-based brain-Interface computer on upper-limb function

**Figure 3:**
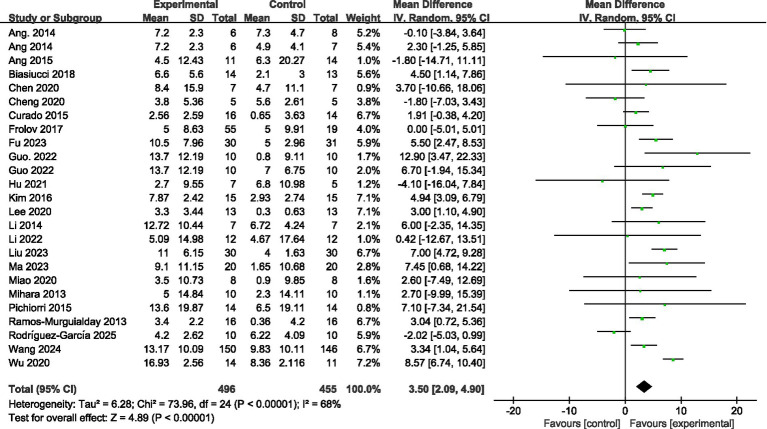
Forest plot indicating the effect of BCI on lower limb function.

As shown in the Figure 3, 23 Studies reported the impact of brain-computer interface-based rehabilitation training on upper limb function. The findings indicated that BCI-based training significantly improved FMA-UE scores compared to the control group, although the heterogeneity was high [WMD = 3.50, 95% CI: (2.09, 4.90), *p* < 0.001, *I*^2^ = 68%].

Regarding age and the severity of sports injuries, the analyses showed that rehabilitation training based on BCI was more effective in individuals under 60 years old, and the treatment had better results in those with severe injuries compared to moderate injuries. Additionally, subgroup analyses of end effectors revealed that BCI-FES and BCI-Robot significantly increased the FMA-UE scores of patients and were more effective than BCI-visual feedback. Concerning training intensity, the schedule with 20–60 min of daily training, 2–5 sessions per week, over a total period of 3 to 4 weeks, proved to be most effective. Specifically, the 20-min and 30-40-min groups showed the greatest improvement. In contrast, durations of 60 min or longer (60–90 min, 90 min) showed no significant effect. Both training frequencies of ≤10 and ≥20 sessions were effective, but extending the total duration to more than 4 weeks did not produce a significant therapeutic benefit (see Supplementary Table 2).

#### 3.4.2 Effect of rehabilitation based brain-interface computer on lower-limb function

**Figure 4:**
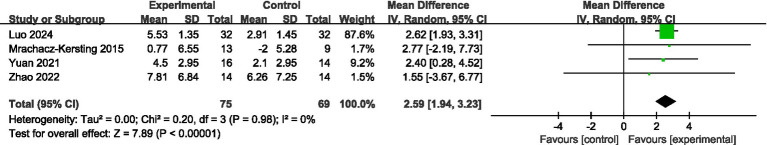
Forest plot indicating the effect of BCI on lower limb function.

Four studies,involving 75 participants across the experimental groups and 144 participants in total, took lower extremity function as one of the outcome indicators. The findings indicated that BCI-based training significantly improved FMA-LE scores [WMD = 2.59, 95%CI (1.94, 3.23), *p <* 0.001]. There was no significant heterogeneity among the included studies (*I*^2^ = 0%, *p* < 0.001) (see Figure 4).

### 3.5 Sensitivity analyses

**Figure 5:**
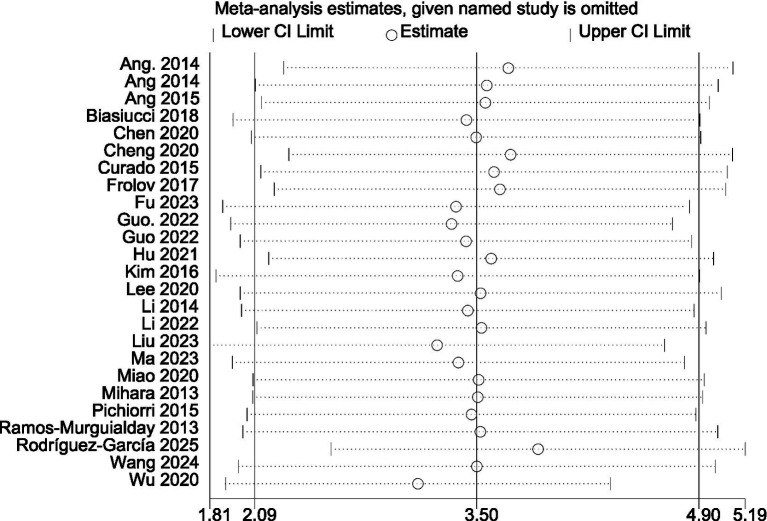
Sensitivity analysis of included studies in upper limb function.

**Figure 6:**
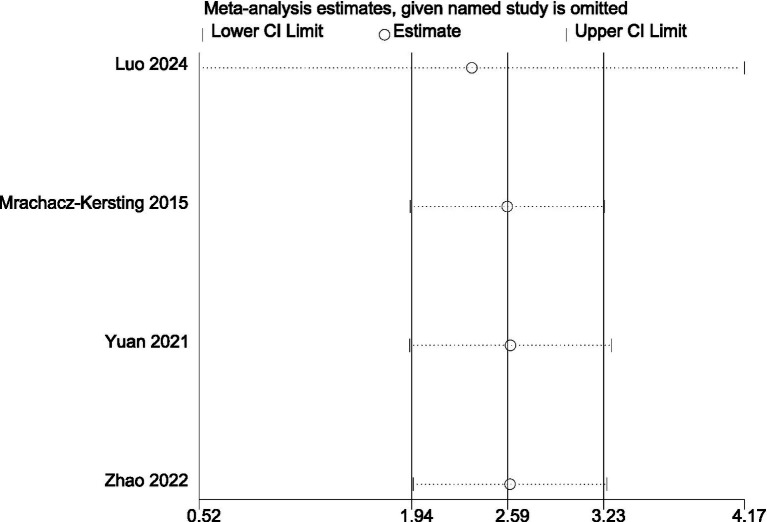
Sensitivity analysis of included studies in lower limb function.

Leave-one-out sensitivity analyses was conducted to evaluate the impact of a single study on the overall results. The results showed that outcomes for upper and lower limb function remained consistent with the original results after excluding any single article, indicating that the results obtained were robust (see Figures 5, 6).

### 3.6 Publication bias and heterogeneity

Publication bias in the included studies was evaluated through funnel plots and Egger’s test. The funnel plots for upper limb and lower limb function appear to be symmetrical, suggesting that publication bias may not be present (see Supplementary Figures 1, 2). At the same time, the Egger test did not find significant evidence of publication bias for FMA-UE (*p* = 0.344) and FMA-LE (*p* = 0.335).

### 3.7 Subgroup analyses

The analyses was conducted based on predefined parameters in the protocol, including age, stroke stage, functional severity, feedback method, and training intensity. Drawing on previous research, we further categorised training intensity into session duration, weekly training frequency, total number of training sessions, and training duration.

## 4 Discussion

The results of the meta-analyses further confirmed that the BCI-based training significantly improves upper limb motor function recovery after stroke and is valuable for lower limb functional rehabilitation. The subgroup analyses results of upper limb outcomes revealed that BCI-based rehabilitation training enhances the therapeutic effects on upper limb function in patients with subacute and chronic stroke, with consistently positive and stable results. There were slight differences in outcomes among the various types of BCIs, with BCI-FES and BCI-Robot showing significant improvements. However, the analyses of the BCI-visual feedback subgroup did not reveal significant effects. Additionally, the analyses suggested that an intervention period of less than 4 weeks may be more conducive to promoting the recovery of upper limb motor function.

Research indicates that BCIs may promote limb function recovery through multiple mechanisms. Firstly, BCIs can enhance synaptic connections. In traditional rehabilitation training, there frequently exists a delay, misalignment, or passive correlation between a patient’s motor intent and actual sensory feedback, making it difficult to meet the “co-firing” requirement of the Hebbian plasticity principle (56). In contrast, BCIs decode cortical signals related to motor imagery or intent in real time and convert them into immediate external feedback. In this process, the firing of cortical neurons and incoming signals achieve precise synchronization at the millisecond level—a core requirement for spatiotemporal-dependent plasticity (STDP) (57, 58). When the presynaptic neuron is repeatedly activated within tens of milliseconds before the postsynaptic neuron fires, long-term potentiation (LTP) occurs at the synapse. If the timing is misaligned, this may induce long-term depression (LTD) or even render the process ineffective. Each BCI training session accomplishes a precise temporal pairing of “command-feedback,” thereby consistently reinforcing the synaptic efficacy of the involved circuit. This also explains why functional improvements can be observed in patients during the chronic phase of stroke. Secondly, BCI can promote neural network reorganization. In post-stroke patients, the excitatory balance between the two cerebral hemispheres is disrupted (59, 60); the unaffected hemisphere becomes overactive, interfering with normal neural coordination and consequently hindering the recovery of motor function. By activating neural networks comprising the ipsilateral premotor cortex, contralateral primary motor cortex, and supplementary motor area, BCI promotes neuronal reorganisation, normalising the excitatory balance between the two hemispheres. Furthermore, BCI training enhances the excitability of the corticospinal tract (55) and improves cortico-muscular coherence in the contralateral motor cortex (61), thereby improving motor function. On this basis, BCIs establishes a closed-loop environment conducive to efficient motor learning. Patients can receive multimodal feedback immediately after each movement attempt. This high-frequency, low-latency enhanced feedback helps patients develop accurate internal motor representations more quickly.

Functional recovery in both the upper and lower limbs is facilitated by neural plasticity mechanisms, although there are some differences. Post-stroke impairments in the lower limb frequently present as difficulties in gait initiation, diminished weight-bearing capacity, and coordination deficits. These complex tasks generally necessitate a higher volume of repetitive practice and richer neural feedback, thus imposing greater demands on the integration of neural circuits, which may result in less favorable intervention outcomes (62). Lower-limb motor control engages a broader neural network, including the supplementary motor area (SMA), the cingulate motor area, and the lateral pathways of the corticospinal tract. Additionally, the signal-to-noise ratio of EEG signals related to lower-limb motor imagery is lower than that associated with the upper limbs, thereby significantly increasing the difficulty of feature extraction and decoding (63, 64). As a consequence, BCI training faces greater challenges in lower-limb rehabilitation. In this meta-analyses, only four studies on lower-limb rehabilitation were included. Although the results indicate functional improvements, the effect sizes were modest, and the findings exhibited a lack of consistency. Further high-quality studies are required to furnish more reliable evidence.

Although the benefits of BCIs for upper limb motor function have been confirmed, its effects in clinical practice are often influenced by various factors. Therefore, we conducted a subgroup analyses to identify the potential factors that affect the treatment outcome. Subgroup analyses targeting the degree of motor impairment (65) revealed that BCI rehabilitation training significantly improved the outcomes of stroke patients with varying degrees of impairment. Patients with severe injuries showed greater improvement in FMA-UE. This phenomenon may be related to differences in the two groups’ neural mechanisms. Patients with severe injuries often have “functionally silent” cortical motor areas, and BCI can activate dormant neural circuits more effectively through closed-loop stimulation to promote synaptic remodeling. In contrast, patients with moderate-to-severe injuries may already have partially activated compensatory mechanisms in undamaged neural pathways, resulting in limited additional gains from BCIs. Additionally, patients with severe injuries have a more urgent need for rehabilitation training and are more actively involved due to severe limitations in daily activities, which enhances the efficacy of BCIs.

Further subgroup analyses of the end-effectors revealed that different types of BCIs have varying effects on upper limb dysfunction. Among them, both BCI-FES and BCI-Robot could significantly improve the upper limb motor function of patients, and were superior to the control group, while BCI-Visual feedback showed negative results. This difference may be due to the synergistic effect of the multimodal feedback mechanisms. Ono et al. (66) conducted a pilot study showing that external devices providing proprioceptive feedback are often more effective than visual feedback in clinical outcomes, suggesting that external devices may affect the effect of brain-computer interaction. Specifically, BCI-FES activates muscle contraction through electrical stimulation and simultaneously triggers tactile and proprioceptive signals (67–69), thereby enhancing activation in the sensorimotor cortex. This process does not excessively tax the participant’s attention or use up visual or auditory channels, preventing any extra disruption to learning. BCI-robot, meanwhile, provides precise joint motor assistance and mechanical feedback, both of which can enhance the functional connectivity of the sensorimotor cortex (70, 71). In contrast, BCI-visual feedback relies on the visual cortex to transmit motor intentions indirectly and lacks direct proprioceptive stimulation. This single sensory input mode may not be sufficient to induce effective neural remodeling (72). Thus, BCI-FES seems to be a more promising external feedback modality. However, no clinical study has directly compared the effectiveness of different feedback modalities, and further research is needed.

There is a critical window of opportunity for the restoration of neurological function following a stroke, and the subacute stage is considered as the best period for enhanced neural plasticity and functional recovery. Consistent with the findings of numerous previous studies, this study found significant improvement in upper-limb function among patients in the subacute phase (73, 74). By establishing an “intention-feedback” closed-loop system, BCI technology can more efficiently strengthen synaptic connections between damaged cortical and spinal pathways during the subacute phase (75). Notably, upper limb function showed significant improvement in patients during the chronic phase. This indicates that even when stroke patients approach a recovery plateau, brain-computer interface technology can still support functional recovery by persistently stimulating partially intact neural pathways and promoting compensatory movement patterns. Regarding training dosage, engaging in 20 to 60 min daily, 2 to 5 times weekly, over a period of 3 to 4 weeks seems to result in more effective training outcomes. One reason for this may be that insufficient daily training duration prevents meaningful improvements, while excessive training increases fatigue and mood changes (37, 76, 77). It should be noted that the training dose regimen outlined in this study is not meant to serve as a final guideline. Instead, it highlights a potentially optimal therapeutic dose based on current data. Further validation of this training dose will be necessary in future studies.

By systematically evaluating the clinical efficacy of three mainstream BCI paradigms, this study provides a reference rehabilitation plan for stroke patients. This study also has certain limitations. Firstly, compared with upper-limb studies, the number of studies using BCI to address lower-limb motor impairments is extremely limited (*n* = 4). Although sensitivity analyses and Egger’s test were employed to assess robustness, the small size of the included studies resulted in low statistical power, making it difficult to reliably rule out potential publication bias or small-sample effects. This limitation subsequently constrains the reliability of our conclusions. Secondly, the majority of studies did not provide follow-up data, which prevents evaluation of the long-term effectiveness of motor imagery-based brain-computer interfaces in enhancing patients’ upper-limb function. Finally, only English-language studies were included, which may limit the comprehensiveness of the findings.

Future research should prioritize improving BCI accuracy and optimizing and standardizing the design of BCI-based training research. At the same time, long-term, large-sample clinical verification is needed. This not only clarifies the most effective brain-computer interface intervention plan for limb function rehabilitation but also provides more precise and personalized rehabilitation strategies for stroke patients. In addition, subsequent research needs to directly compare the actual effects of the three types of BCI end effectors to clearly define their applicable scenarios and optimization directions, thereby providing a higher-level evidence-based basis for clinical decision-making.

## 5 Conclusion

The existing evidence obtained in this study supports the use of BCI-based rehabilitation methods to improve the motor function of stroke patients. The combination of BCI with FES appears particularly promising, catering to patients in both the subacute and chronic phases. A training intensity of 20–60 min per day, 2–5 sessions per week, for 3–4 weeks may be most recommended. This study provides evidence-based evidence for the rehabilitation path of motor dysfunction after stroke, and also offers strategic references for therapists and doctors.

## Data Availability

The original contributions presented in the study are included in the article/Supplementary material, further inquiries can be directed to the corresponding author.
