# Supplementary material for: The effect of rehabilitation training based on brain-computer interface on limb function in stroke patients: a systematic review and meta-analyses
Source: Front Neurol. 2026 Jul 1;17:1750875. doi: 10.3389/fneur.2026.1750875 (PMC13368990; doi:10.3389/fneur.2026.1750875)
Supplement: Supplementary file 1 [file Supplementary_file_1.docx]

**Supplementary Fig.1:** **Funnel plot for the association between BCI and upper limb function**

**.**

**Supplementary Fig.2:** **Funnel plot for the association between BCI and lower limb function**

**Supplementary Table.1: Search strategy**

| Database name | Search strategies: key words and how these were combined in the search | Laster update | Number of studies identified |
| --- | --- | --- | --- |
| PubMed | ((("Stroke"[Mesh]) OR ((((((((((((((((Cerebrovascular Accident*[Title/Abstract]) OR (Cerebral Stroke*[Title/Abstract])) OR (Stroke*, Cerebral[Title/Abstract])) OR (Cerebrovascular Apoplexy[Title/Abstract])) OR (Apoplexy, Cerebrovascular[Title/Abstract])) OR (Vascular Accident*, Brain[Title/Abstract])) OR (Brain Vascular Accident*[Title/Abstract])) OR (Cerebrovascular Stroke*[Title/Abstract])) OR (Stroke*, Cerebrovascular[Title/Abstract])) OR (Apoplexy[Title/Abstract])) OR (CVA (Cerebrovascular Accident[Title/Abstract]))) OR (CVAs (Cerebrovascular Accident[Title/Abstract]))) OR (Stroke*, Acute[Title/Abstract])) OR (Acute Stroke*[Title/Abstract])) OR (Cerebrovascular Accident*, Acute[Title/Abstract])) OR (Acute Cerebrovascular Accident*[Title/Abstract]))) AND (("Brain-Computer Interfaces"[Mesh]) OR ((((((((((((((Brain-Computer Interface[Title/Abstract]) OR (Brain Computer Interface*[Title/Abstract])) OR (Interface*, Brain-Computer[Title/Abstract])) OR (Neural Interface Technolog*[Title/Abstract])) OR (Interface Technolog*, Neural[Title/Abstract])) OR (Technolog*, Neural Interface[Title/Abstract])) OR (Brain-Machine Interface*[Title/Abstract])) OR (Interface*, Brain-Machine[Title/Abstract])) OR (Brain Machine Interface*[Title/Abstract])) OR (Interface*, Brain Machine[Title/Abstract])) OR (Machine Interface*, Brain[Title/Abstract])) OR (BCI-FES[Title/Abstract])) OR (BCI-robot[Title/Abstract]))))) AND (((randomized controlled trial[Publication Type]) OR (controlled clinical trial[Publication Type])) OR (RCT[Title/Abstract] OR Randomized[Title/Abstract] OR clinical trial[Title/Abstract] OR Randomly[Title/Abstract] OR Trial[Title/Abstract] OR Control[Title/Abstract] OR Placebo[Title/Abstract] OR groups[Title/Abstract])) | 29th  8  2025 | 181 |
| Scopus | ( ( ( TITLE-ABS-KEY ( "Stroke*" ) OR TITLE-ABS-KEY ( "Cerebrovascular Accident*" ) OR TITLE-ABS-KEY ( "Cerebral Stroke*" ) OR TITLE-ABS-KEY ( "Stroke*, Cerebral" ) OR TITLE-ABS-KEY ( "Cerebrovascular Apoplexy" ) OR TITLE-ABS-KEY ( "Apoplexy, Cerebrovascular" ) OR TITLE-ABS-KEY ( "Vascular Accident*, Brain" ) OR TITLE-ABS-KEY ( "Brain Vascular Accident*" ) OR TITLE-ABS-KEY ( "Cerebrovascular Stroke*" ) OR TITLE-ABS-KEY ( "Stroke*, Cerebrovascular" ) OR TITLE-ABS-KEY ( "Apoplexy" ) OR TITLE-ABS-KEY ( "CVA (Cerebrovascular Accident)" ) OR TITLE-ABS-KEY ( "CVAs (Cerebrovascular Accident)" ) OR TITLE-ABS-KEY ( "Stroke*, Acute" ) OR TITLE-ABS-KEY ( "Acute Stroke*" ) OR TITLE-ABS-KEY ( "Cerebrovascular Accident*, Acute" ) OR TITLE-ABS-KEY ( "Acute Cerebrovascular Accident*" ) ) ) AND ( ( TITLE-ABS-KEY ( "Brain-Computer Interface" ) OR TITLE-ABS-KEY ( "Brain Computer Interface*" ) OR TITLE-ABS-KEY ( "Interface*, Brain-Computer*" ) OR TITLE-ABS-KEY ( "Neural Interface Technolog*" ) OR TITLE-ABS-KEY ( "Interface Technolog*, Neural" ) OR TITLE-ABS-KEY ( "Technolog*, Neural Interface" ) OR TITLE-ABS-KEY ( "Brain-Machine Interface*" ) OR TITLE-ABS-KEY ( "Interface*, Brain-Machine" ) OR TITLE-ABS-KEY ( "Brain Machine Interface*" ) OR TITLE-ABS-KEY ( "Interface*, Brain Machine" ) OR TITLE-ABS-KEY ( "Machine Interface*, Brain" ) OR TITLE-ABS-KEY ( "BCI-FES" ) OR TITLE-ABS-KEY ( "BCI-robot" ) ) ) ) AND ( ( ( TITLE-ABS-KEY ( "randomized controlled trial" ) OR TITLE-ABS-KEY ( "rct" ) OR TITLE-ABS-KEY ( "controlled clinical trial" ) OR TITLE-ABS-KEY ( "randomized" ) OR TITLE-ABS-KEY ( "clinical trial" ) OR TITLE-ABS-KEY ( "randomly" ) OR TITLE-ABS-KEY ( "trial" ) OR TITLE-ABS-KEY ( "control" ) OR TITLE-ABS-KEY ( "placebo" ) OR TITLE-ABS-KEY ( "groups " ) ) ) ) | 29th  8  2025 | 1033 |
| Web of science | #1((((((((((((((((TS=(Stroke*)) OR TS=(Cerebrovascular Accident*)) OR TS=(Cerebral Stroke*)) OR TS=(Stroke*, Cerebral)) OR TS=(Cerebrovascular Apoplexy)) OR TS=(Apoplexy, Cerebrovascular)) OR TS=(Vascular Accident*, Brain)) OR TS=(Brain Vascular Accident*)) OR TS=(Cerebrovascular Stroke*)) OR TS=(Stroke*, Cerebrovascular)) OR TS=(Apoplexy)) OR TS=(CVA (Cerebrovascular Accident))) OR TS=(CVAs (Cerebrovascular Accident))) OR TS=(Stroke*, Acute)) OR TS=(Acute Stroke*)) OR TS=(Cerebrovascular Accident*, Acute)) OR TS=(Acute Cerebrovascular Accident*) 763981  #2 ((((((((((((TS=(Brain-Computer Interface)) OR TS=(Brain Computer Interface*)) OR TS=(Interface*, Brain-Computer)) OR TS=(Neural Interface Technolog*)) OR TS=(Interface Technolog*, Neural)) OR TS=(Technolog*, Neural Interface)) OR TS=(Brain-Machine Interface*)) OR TS=(Interface*, Brain-Machine)) OR TS=(Brain Machine Interface*)) OR TS=(Interface*, Brain Machine)) OR TS=(Machine Interface*, Brain)) OR TS=(BCI-FES)) OR TS=(BCI-robot) 48189  #4 ((((((((((TS=(randomized controlled trial)) OR TS=(RCT)) OR TS=(controlled clinical trial)) OR TS=(Randomized)) OR TS=(clinical trial)) OR TS=(Randomly)) OR TS=(Trial))) OR TS=( Control)) OR TS=(Placebo)) OR TS=(groups) 23008696  #1 AND #2 AND # 1085 | 29th  8  2025 | 2277 |
| Cochrane library | #1 MeSH descriptor: [Stroke] explode all trees 17720  #2 (Stroke):ti,ab,kw OR (Strokes):ti,ab,kw OR (Apoplexy):ti,ab,kw OR (Cerebrovascular Apoplexy):ti,ab,kw OR (Apoplexy, Cerebrovascular):ti,ab,kw OR (CVA (Cerebrovascular Accident)):ti,ab,kw OR (CVAs (Cerebrovascular Accident)):ti,ab,kw OR (Cerebrovascular Accident):ti,ab,kw OR (Cerebrovascular Accidents):ti,ab,kw OR (Cerebrovascular Stroke):ti,ab,kw OR (Cerebrovascular Strokes):ti,ab,kw OR (Vascular Accident, Brain):ti,ab,kw OR (Vascular Accidents, Brain):ti,ab,kw OR (Brain Vascular Accident):ti,ab,kw OR (Brain Vascular Accidents):ti,ab,kw OR (Stroke, Cerebrovascular):ti,ab,kw OR (Strokes, Cerebrovascular):ti,ab,kw OR (Cerebral Stroke):ti,ab,kw OR (Cerebral Strokes):ti,ab,kw OR (Stroke, Cerebral):ti,ab,kw OR (Strokes, Cerebral):ti,ab,kw OR (Cerebrovascular Accident, Acute):ti,ab,kw OR (Cerebrovascular Accidents, Acute):ti,ab,kw OR (Acute Stroke):ti,ab,kw OR (Acute Strokes):ti,ab,kw OR (Acute Cerebrovascular Accident):ti,ab,kw OR (Acute Cerebrovascular Accidents):ti,ab,kw OR (Stroke, Acute):ti,ab,kw OR (Strokes, Acute):ti,ab,kw 79535  #3 #1 OR #2 80061  #4 MeSH descriptor: [Brain-Computer Interfaces] explode all trees 113  #5 (Interface, Brain-Computer):ti,ab,kw OR (Technologies, Neural Interface):ti,ab,kw OR (Neural Interface Technology):ti,ab,kw OR (Interface Technology, Neural):ti,ab,kw OR (Brain Computer Interface):ti,ab,kw OR (Brain Computer Interfaces):ti,ab,kw OR (Neural Interface Technologies):ti,ab,kw OR (Interface Technologies, Neural):ti,ab,kw OR (Technology, Neural Interface):ti,ab,kw OR (Interfaces, Brain-Computer):ti,ab,kw OR (Brain-Computer Interface):ti,ab,kw OR (Interfaces, Brain Machine):ti,ab,kw OR (Interface, Brain Machine):ti,ab,kw OR (Interface, Brain-Machine):ti,ab,kw OR (Machine Interfaces, Brain):ti,ab,kw OR (Brain-Machine Interfaces):ti,ab,kw OR (Interfaces, Brain-Machine):ti,ab,kw OR (Brain Machine Interfaces):ti,ab,kw OR (Machine Interface, Brain):ti,ab,kw OR (Brain Machine Interface):ti,ab,kw OR (Brain-Machine Interface):ti,ab,kw OR (BCI-FES):ti,ab,kw OR (BCI-robot):ti,ab,kw 620  #6 #4 OR #5 620  #7 (randomized controlled trial):ti,ab,kw OR (RCT):ti,ab,kw OR (controlled clinical trial):ti,ab,kw OR (Randomized):ti,ab,kw OR (clinical trial):ti,ab,kw OR (Randomly):ti,ab,kw OR (Trial):ti,ab,kw OR (Control):ti,ab,kw OR (Placebo):ti,ab,kw OR (groups ):ti,ab,kw 1762119  #8 #3 AND #6 AND #7 229 | 29th  8  2025 | 229 |
| Embase | #1**'cerebrovascular accident'**/exp 486423  #2 'accident, cerebrovascular':ti,ab,kw OR 'acute cerebrovascular lesion':ti,ab,kw OR 'acute focal cerebral vasculopathy':ti,ab,kw OR 'acute stroke':ti,ab,kw OR 'apoplectic stroke':ti,ab,kw OR apoplexia:ti,ab,kw OR apoplexy:ti,ab,kw OR 'blood flow disturbance, brain':ti,ab,kw OR 'brain accident':ti,ab,kw OR 'brain attack':ti,ab,kw OR 'brain blood flow disturbance':ti,ab,kw OR 'brain insult':ti,ab,kw OR 'brain insultus':ti,ab,kw OR 'brain vascular accident':ti,ab,kw OR 'cerebral apoplexia':ti,ab,kw OR 'cerebral insult':ti,ab,kw OR 'cerebral stroke':ti,ab,kw OR 'cerebral vascular accident':ti,ab,kw OR 'cerebral vascular insufficiency':ti,ab,kw OR 'cerebro vascular accident':ti,ab,kw OR 'cerebrovascular arrest':ti,ab,kw OR 'cerebrovascular failure':ti,ab,kw OR 'cerebrovascular injury':ti,ab,kw OR 'cerebrovascular insufficiency':ti,ab,kw OR 'cerebrovascular insult':ti,ab,kw OR 'cerebrum vascular accident':ti,ab,kw OR 'cryptogenic stroke':ti,ab,kw OR cva:ti,ab,kw OR 'insultus cerebralis':ti,ab,kw OR 'ischaemic seizure':ti,ab,kw OR 'ischemic seizure':ti,ab,kw OR stroke:ti,ab,kw OR 'thrombotic stroke':ti,ab,kw OR 'cerebrovascular accident':ti,ab,kw 577560  #3 #1 OR #2 701237  #4 'brain computer interface'/exp 11110  #5 'bci system':ti,ab,kw AND 'brain-computer interface':ti,ab,kw OR 'bci-controlled neuroprosthetic':ti,ab,kw OR 'brain computer interface system':ti,ab,kw OR 'brain computer interfaces':ti,ab,kw OR 'brain computing interface':ti,ab,kw OR 'brain machine interface':ti,ab,kw OR 'brain-computer interface':ti,ab,kw OR 'brain-computer interfaces':ti,ab,kw OR 'brain-machine interface':ti,ab,kw OR 'cerebellum-machine interface':ti,ab,kw OR 'cerebral-computer interfaces':ti,ab,kw OR 'direct neural interface':ti,ab,kw OR 'mind-machine interface':ti,ab,kw OR 'brain computer interface':ti,ab,kw OR 'bci fes':ti,ab,kw OR 'bci robot':ti,ab,kw OR 'brain-computer interfacing system':ti,ab,kw 11342  #6 #4 OR #5 13909  #7 'randomized controlled trial'/exp OR 'randomized controlled trial (topic)'/exp OR 'randomized controlled trial':ti,ab,kw OR 'rct':ti,ab,kw OR 'controlled clinical trial':ti,ab,kw OR 'randomized':ti,ab,kw OR 'clinical trial':ti,ab,kw OR 'randomly':ti,ab,kw OR 'trial':ti,ab,kw OR 'control':ti,ab,kw OR 'placebo':ti,ab,kw OR 'groups':ti,ab,kw 8938913  #8 #3 AND #6 AND #8 703 | 29th  8  2025 | 703 |

**Supplementary Table 2: Results of subgroup analysis of included studies in the meta-analysis**

|  | | | | |  |
| --- | --- | --- | --- | --- | --- |
| **Groups** | n | **FMA-UE** | | |  |
|  |  | SMD^‡^(95%CI^§^) | I^2^(%) | P-value |  |
| **Stroke type** |  |  |  |  |  |
| subacute | 6 | 6.18 [3.64, 8.73] | 61 | 0.000 |  |
| chronic | 13 | 3.04 [1.81, 4.27] | 32 | 0.000 |  |
| **Base FMA-UE scores** |  |  |  |  |  |
| 10~20 scores | 8 | 5.13 [2.34, 7.92] | 76 | 0.000 |  |
| 20~30 scores | 14 | 3.07 [1.97, 4.18] | 13 | 0.310 |  |
| 30~40 scores | 2 | 6.92 [4.67, 9.17] | 0 | 0.660 |  |
| **Age** |  |  |  |  |  |
| ≤60 years old | 21 | 3.31 [2.07, 4.55] | 53 | 0.000 |  |
| ＞60 years old | 4 | 4.76 [-1.53, 11.06] | 78 | 0.140 |  |
| **BCI-type** |  |  |  |  |  |
| BCI-FES | 7 | 4.52 [3.27, 5.76] | 29 | 0.000 |  |
| BCI-Robot | 15 | 2.98 [0.70, 5.26] | 77 | 0.010 |  |
| BCI-visual feedback | 3 | 1.23 [-6.23, 8.68] | 0 | 0.750 |  |
| **Training time per day** |  |  |  |  |  |
| <20 minutes | 2 | 2.96 [-5 29, 11.22] | 0 | 0.480 |  |
| 20 minutes | 2 | 6.87 [4.63, 9.11] | 0 | 0.000 |  |
| 30-40 minutes | 8 | 3.82 [2.55, 5.09] | 20 | 0.000 |  |
| 60 minutes | 10 | 3.59 [0 .91, 6.27] | 83 | 0.009 |  |
| 60-90 minutes | 1 | 6.00 [-2.35, 14.35] | - | 0.160 |  |
| 90 minutes | 2 | -1.80 [-6.64, 3.04] | 0 | 0.470 |  |
| **Training sessions per week** |  |  |  |  |  |
| 2-3 sessions | 13 | 3.63 [1.60, 5.66] | 55 | 0.001 |  |
| 5 sessions | 11 | 3.28 [1.32, 5.25] | 69 | 0.000 |  |
| **Total training sessions** |  |  |  |  |  |
| <10 sessions | 7 | 4.54 [1.66, 7.43] | 21 | 0.002 |  |
| 10-20 sessions | 9 | 3.08 [0.71, 5.45] | 57 | 0.010 |  |
| ≥20 sessions | 8 | 3.54 [1.24, 5.85] | 85 | 0.003 |  |
| **Training duration** |  |  |  |  |  |
| ≤ 2 weeks | 6 | 4.86 [0.65, 9.08] | 34 | 0.020 |  |
| 3-4 weeks | 10 | 4.28 [2.58, 5.99] | 70 | 0.000 |  |
| >4 weeks | 6 | 1.08 [-1.43, 3.59] | 56 | 0.400 |  |
| ^‡^ SMD: standardized mean difference; ^§^CI: confidence interval;  FMA-UE: Fugl-Meyer Assessment upper-extremity | | | | |  |
